# Numerical Investigation of Differential Speed Coupling Mechanisms in a 600 L Eccentric Four-Shaft Mixer for High-Viscosity Polyurethane Adhesives

**DOI:** 10.3390/ma19153285

**Published:** 2026-08-03

**Authors:** Yongli Luo, Long Fan, Bin He, Xing Fan, Facheng Qiu, Renlong Liu

**Affiliations:** 1 College of Chemistry and Chemical Engineering, Chongqing University, Chongqing 400044, China; 2 College of Chemistry and Chemical Engineering, Chongqing University of Technology, Chongqing 400054, China

**Keywords:** polyurethane adhesive, multi-axis stirring, computational fluid dynamics, differential ratio, chaotic mixing, scale-up, high-viscosity fluid

## Abstract

Polyurethane adhesives often suffer from high viscosity, poor flowability, and limited mass transfer efficiency, making it challenging for conventional single-shaft or twin-shaft mixing systems to achieve uniform mixing in large-capacity equipment. This study investigates the differential speed coupling mechanism of a 600 L eccentric four-shaft mixer for high-viscosity polyurethane adhesive systems using computational fluid dynamics (CFD). The effects of different differential speed ratios on flow field evolution and mixing performance were systematically analyzed through multiple indicators, including power consumption per unit volume, velocity variation coefficient, effective mixing region ratio, vortex core coverage, and maximum Lyapunov exponent (LLE). Among the investigated conditions, the 50:100 differential speed ratio exhibited a more favorable circulation pattern, forming a closed-loop circulating flow field in the entire reactor, with an effective mixing region accounting for 60%, a velocity variation coefficient of only 0.38, and a power consumption per unit volume as low as 21.2 W/m^3^. A constant speed of 100:100 easily generates dead zones in the interaxial flow field, while a high differential speed of 100:50 leads to excessive disturbances and energy waste. Moderate differential speed can generate large-scale coupled vortices, thereby enhancing mixing through coupled axial transport, radial dispersion, and tangential shear. An eccentric four-shaft mixer combined with reasonable differential speed control can effectively improve flow field uniformity and reduce potential mixing limitations in large-scale polyurethane adhesive systems. Among the three investigated differential speed ratios, the 50:100 condition exhibited the most favorable overall mixing performance. These findings provide numerical insights into the selection of differential speed operating conditions for high-viscosity polyurethane adhesive mixing systems.

## 1. Introduction

Polyurethane adhesives have excellent bonding performance, chemical corrosion resistance and mechanical properties, and have been widely applied in high-end manufacturing fields such as new energy vehicles, rail transportation, aerospace and electronic packaging [1,2,3,4,5]. With the increasing demand for high-performance adhesives, polyurethane adhesive systems are gradually evolving toward higher solid content, enhanced functionality, and increased viscosity. The uniformity of the mixing process during the preparation has become a key issue restricting the improvement of product quality. At the same time, the manufacturing of polyurethane materials is gradually moving towards continuous, automated and intelligent directions, which places higher demands on the mixing efficiency and product consistency in large-scale production processes [6].

The high-viscosity polyurethane system exhibits significant non-Newtonian rheological properties, and the mixing process is highly dependent on mechanical stirring for dispersion and mass transfer [7]. As the scale of the equipment expands from the laboratory to industrialization, the fluid transmission distance increases and the viscosity dissipation intensifies. This often leads to problems such as flow field stratification, near-wall stagnation, bottom deposition, and local dead zones, resulting in a significant decline in mixing efficiency [8,9,10]. These issues may cause poor filler dispersion and concentration fluctuations, ultimately affecting adhesive performance and production stability [11].

In current industrial processes, high-viscosity materials are typically mixed using single-axis, double-axis or planetary-type mixing structures. The single-axis structure is simple but has weak circulation capacity and obvious dead zones. The double-axis and planetary structures can enhance shear and circulation performance, but they still struggle to overcome the problems of flow stratification, insufficient near-wall mixing and high energy consumption in large-scale operations. In recent years, multi-axis mixing technology has gained widespread attention due to its strong synergy of high shear and strong circulation [12,13,14]. Through multi-axis flow field coupling, the transportation and dispersion of materials can be enhanced, and the overall mixing efficiency can be improved. However, for the medium-scale high-viscosity polyurethane system, the evolution law of the flow field and the mixing enhancement mechanism under the regulation of differential ratios still lack systematic research [15].

Therefore, the development of multi-axis mixing systems with coordinated shear and circulation capabilities provides a potential solution for large-scale processing of high-viscosity polyurethane adhesives. An eccentric four-shaft configuration provides additional flexibility in regulating the interaction between axial transport and radial dispersion through independent control of different functional shafts. By coupling high-shear dispersion units with axial transport units, this configuration offers potential advantages for balancing local shear generation and global circulation in highly viscous adhesive processing. However, the relationship between differential speed coupling and mixing enhancement in industrial-scale high-viscosity adhesive systems remains unclear, which motivates the present investigation.

Computational fluid dynamics (CFD) has become an important research method for analyzing complex mixing flow fields, particularly for high-viscosity fluid systems where direct experimental characterization is often difficult. It enables the visualization and quantitative characterization of velocity fields, streamline structures, vortex characteristics, and energy distribution, providing reliable theoretical support for the structural optimization of mixing equipment and the matching of process parameters [9,16,17]. In recent years, CFD investigations of industrial mixing systems have increasingly focused on high-viscosity non-Newtonian fluids, multi-axis mixing configurations, and the coupling effects between structural design and operating parameters. Previous CFD studies on complex industrial mixing equipment, including twin-shaft kneaders, eccentric mixers, and multi-shaft stirred reactors, have demonstrated the capability of numerical methods in predicting flow evolution and optimizing mixing performance [10,12,13,14,15]. In particular, CFD approaches have been widely applied to high-viscosity adhesive systems, where the effects of mixing structures and operating conditions on flow field characteristics and mixing efficiency can be effectively revealed [13,15]. However, despite recent progress in CFD-based analysis of complex mixing systems, the differential speed coupling mechanism between different functional stirring units in large-scale polyurethane adhesive mixers remains insufficiently understood, particularly regarding its influence on flow field organization, energy transfer, and mixing enhancement. Here, the differential speed ratio refers to the relative rotational speed relationship between the axial propulsion shafts and main dispersion shafts, which determines the balance between global circulation and local shear enhancement. Furthermore, recent advances have promoted the integration of CFD with data-driven approaches, such as machine learning, to improve the analysis efficiency and optimization capability of complex flow systems [18,19].

Various types of industrial mixers have been developed to address the challenges associated with high-viscosity material processing. Single-shaft mixers provide simple structures and reliable operation but often suffer from insufficient circulation and dead zones in highly viscous systems. Dual-shaft and planetary mixers can improve shear intensity and mixing efficiency; however, complex flow interactions, high energy consumption, and scale-up limitations remain challenging. Eccentric and multi-shaft mixers further enhance flow disturbance by introducing multiple rotation centers and integrating different mixing functions. Nevertheless, previous studies have mainly focused on mixer structural optimization, flow field characterization, or overall mixing performance evaluation, while the interaction mechanism between different functional stirring units under differential speed operation remains unclear. In particular, the coupling between axial circulation transport and radial dispersion enhancement in large-scale polyurethane adhesive systems has rarely been investigated.

Therefore, despite the increasing application of CFD methods in high-viscosity mixing equipment, the differential speed coupling mechanism between functional stirring units in industrial-scale multi-axis mixers remains insufficiently understood. Previous studies have primarily investigated the effects of mixer geometry, impeller configuration, or individual operating parameters, whereas the synergistic interaction between high-shear dispersion and large-scale circulation under differential speed regulation has received limited attention, particularly in large-capacity polyurethane adhesive systems.

In this work, a 600 L eccentric four-shaft mixing system for high-viscosity polyurethane adhesives was established, consisting of two high-speed dispersion shafts and two axial propulsion shafts with different functional roles. Unlike conventional multi-axis mixing studies, this study focuses on the coupling effect between local shear enhancement and global circulation driven by differential speed matching. The influence of three representative differential speed ratios on flow field evolution, energy consumption characteristics, and mixing enhancement characteristics was comparatively investigated using CFD simulations. Multiple quantitative indicators, including unit volume power consumption, velocity variation coefficient, effective mixing region ratio, vortex core coverage, and maximum Lyapunov exponent (LLE), were integrated to reveal the relationship between differential speed regulation and mixing enhancement mechanisms. The results provide new insights into the operation optimization of industrial-scale multi-axis mixers and offer theoretical guidance for improving the mixing efficiency of high-viscosity polyurethane adhesive systems.

## 2. Numerical Models and Research Methods

### 2.1. Geometric Model Establishment

**Figure 1:**
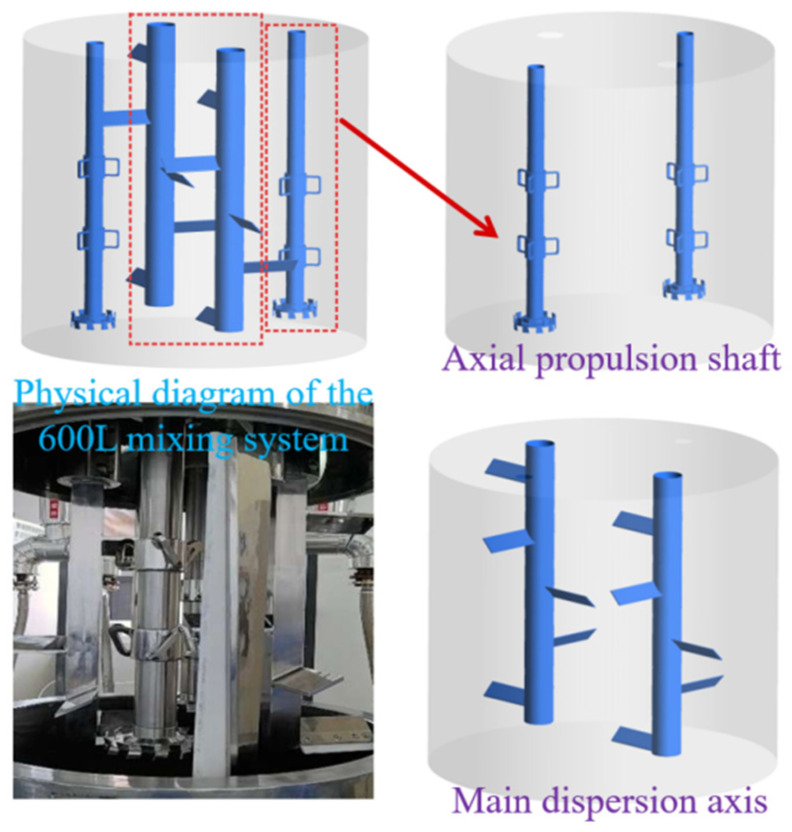
Three-dimensional schematic diagram of a 600 L multi-axis stirring structure. (The red arrow indicates the enlarged view of the axial propulsion shaft and main dispersion axis from the overall stirring system).

To explore the flow field evolution laws and mixing enhancement mechanisms of high-viscosity polyurethane adhesives under different differential ratios, a three-dimensional geometric model of a 600 L eccentric four-axis stirring tank was established, as shown in Figure 1. The stirring device consists of two main dispersing axes and two axial propulsion axes: the main dispersing axes are symmetrically arranged on both sides of the tank center, with an axis distance from the tank center of 0.3 m, an axis diameter of 0.025 m, and a total length of 0.77 m; the axes are equipped with toothed dispersing disks, with a disk thickness of 3 mm, a tooth height of 0.02 m, an outer diameter of 0.12 m, and 18 groups of mixing teeth evenly distributed on the disk surface. Relying on high-speed rotation, local strong shear is generated to achieve filler fragmentation and polymer chain disentanglement. The axial propulsion axes are eccentrically arranged, with an axis distance from the tank center of 0.25 m, an axis diameter of 0.04 m, and a total length of 0.78 m; 5 groups of 45° inclined blades are evenly assembled on the axis, with a single blade width of 0.05 m, a blade length of 0.17 m, a thickness of 0.003 m, an axial spacing of 0.156 m, and an outer edge gap with the tank wall of 0.055 m. This focuses on strengthening the axial fluid circulation within the tank.

The geometric model was established based on the actual structural parameters of the 600 L industrial mixer. Considering the physical characteristics of high-viscosity polyurethane adhesive systems, the liquid phase density was set as 960 kg·m^−3^, and the apparent dynamic viscosity was set as 0.35 kg·(m·s)^−1^ in the numerical simulation. A constant-viscosity approximation was adopted to simplify the complex rheological behavior of polyurethane adhesives and improve numerical convergence. Although practical polyurethane adhesives may exhibit shear-dependent rheological characteristics, the present study mainly focuses on the influence of differential speed coupling on the overall flow field structure and mixing behavior.

Compared with the traditional symmetrical arrangement of stirring structures, the eccentric layout can enhance the interaction of flow fields between the axes and the exchange of fluids, effectively weakening the problems of flow stratification, near-wall stagnation, and local dead zones commonly found in large-capacity high-viscosity systems. The two types of stirring axes function complementarily, through the coupling effect of local shear and global circulation, providing a structural basis for improving the mixing uniformity of the adhesive throughout the tank.

### 2.2. Grid Division

Based on the geometric structure characteristics of the 600 L four-axis mixing tank and the flow characteristics of high-viscosity fluids, the ANSYS Workbench 2022 R1 software was used to complete the mesh division of the entire flow field. Considering the complex structure of the mixing system and the large local flow gradients, a combined approach of structured and unstructured meshes was adopted for discretization processing.

**Figure 2:**
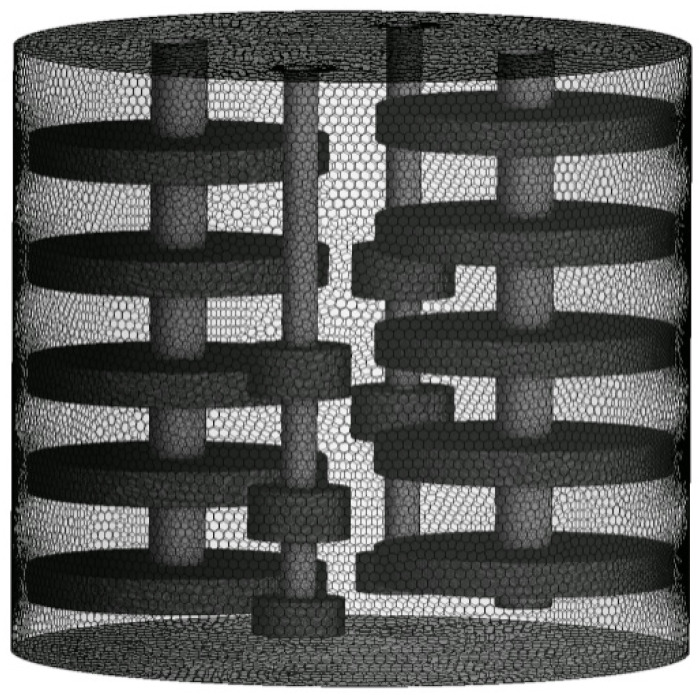
Grid division diagram of a 600 L multi-axis stirred tank.

To improve the calculation accuracy, local mesh densification was carried out in the area near the dispersing disk, the area of axial coupling interaction, and the near-wall area. Specifically, the high shear flow characteristics near the dispersing disk were focused on, the axial area was used to represent the multi-axis coupled flow field structure, and the near-wall area was used to enhance the resolution of the boundary layer flow. Eventually, approximately 25 million calculation units were generated to balance the calculation accuracy and efficiency, as shown in Figure 2.

### 2.3. Grid Independence Verification

**Figure 3:**
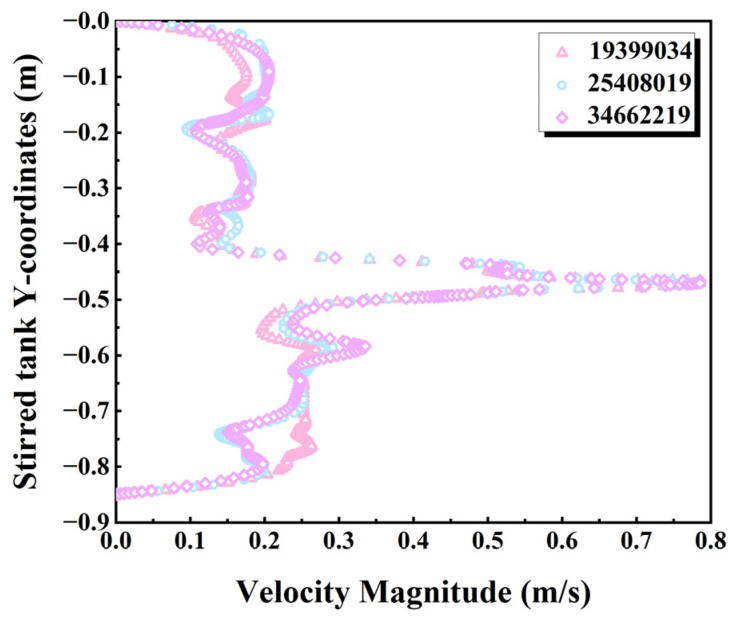
The effect of grid number on velocity distribution.

To ensure that the numerical calculation results are not affected by the number of grids, the grid independence verification was conducted for the established 600 L eccentric four-axis mixing model. Considering the significant changes in the flow field near the mixing paddle and the large velocity gradients in the near-wall region, a hybrid grid partitioning strategy combining structured and unstructured grids was adopted for the model. Local mesh densification was performed in the main dispersion disk area, the inter-axis coupling area, and the near-wall region. Under typical operating conditions, velocity sampling lines were established along the interior of the model, with the sampling line positions set at x = 0.4 m and z = 0 m, and continuous sampling along the y direction from −0.85 m to 0 m. The velocity magnitude was used as the evaluation index, and the velocity distributions under different grid numbers were compared and analyzed. The results are shown in Figure 3.

As shown in Figure 3, the overall trend of the velocity distribution curves remains consistent under different grid conditions. As the number of grids increases, the curves gradually converge. When the number of grids reaches 25,408,019, further increasing the number of grids has a relatively small impact on the velocity distribution, and the calculation results basically no longer show significant changes. This indicates that the numerical simulation results have good grid independence. Considering both calculation accuracy and cost, this paper finally selects 25,408,019 calculation units as the calculation grid for subsequent numerical simulations, which is used to analyze the velocity field, streamline structure, power consumption characteristics, and chaotic mixing characteristics under different differential ratios [20].

In addition to the velocity distribution, the sensitivity of torque and unit volume power consumption to mesh resolution was further examined. The calculated torque values of the stirring shafts and the corresponding power consumption showed negligible variation when the mesh number increased from the intermediate mesh to the final mesh. The relative deviation of torque and power consumption between the selected mesh and the finer mesh remained within an acceptable range, indicating that the selected mesh was sufficient for capturing the main hydrodynamic characteristics of the mixing system.

Moreover, local mesh refinement was specifically applied in regions with strong velocity gradients, including the dispersion disk area, inter-axis interaction region, and near-wall region. This strategy ensured adequate resolution of the high-shear zones and flow coupling structures that are critical for the subsequent evaluation of mixing performance indicators.

### 2.4. Numerical Model Assessment

To assess the numerical performance of the CFD model, grid independence analysis and physical consistency evaluation of key hydrodynamic parameters were performed. Since direct experimental measurement of the internal flow field in a 600 L high-viscosity polyurethane adhesive system is highly challenging, the numerical results were assessed based on mesh independence and the consistency of predicted shaft torque and unit volume power consumption under different differential speed ratios [17,21]. By adopting uniform physical parameters, boundary conditions and solution settings, the torques and unit volume power consumption under three typical working conditions (with the main dispersion axis velocity ratio of 50:100, 100:100 and 100:50) for the axial propulsion axis were calculated respectively, and the variation patterns were analyzed, as summarized in Table 1.

**Figure 4:**
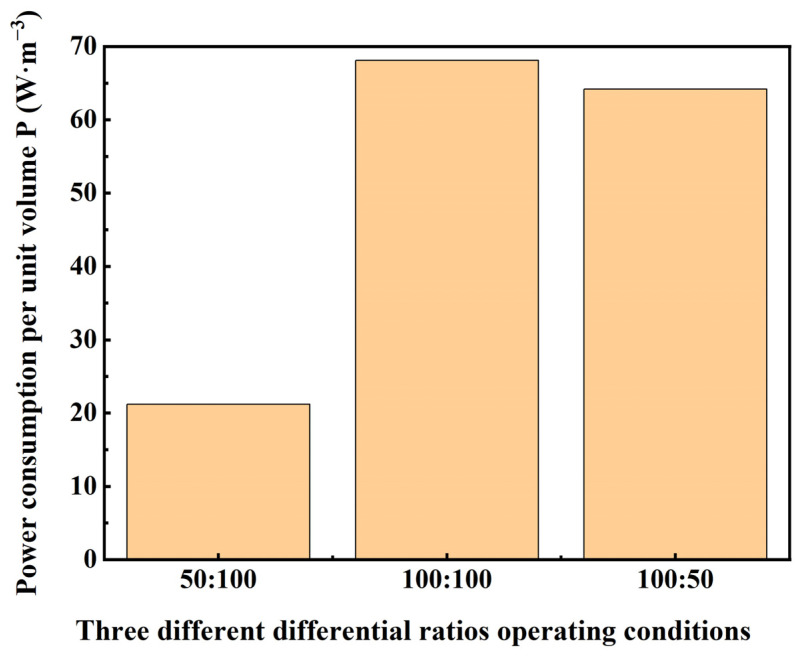
Bar chart of power consumption per unit volume under different differential speed ratios.

From Table 1 and Figure 4, it can be seen that the variation pattern of unit volume power consumption under different differential ratios is 100:100 > 100:50 > 50:100. Among them, the unit volume power consumption under the constant-speed condition is the highest, at 68.1 W/m^3^, followed by the high-differential speed condition, at 64.2 W/m^3^, and the lowest under the low-differential speed condition, at only 21.2 W/m^3^. At the same time, the axial thrust torque of each condition is significantly higher than the main dispersion torque, indicating that the propulsion axis bears the main role in the circulation transport, while the main dispersion axis mainly provides a local high shear effect. This result is consistent with the structural design of the eccentric four-axis mixer and the mixing mechanism of high-viscosity fluids.

Further analysis shows that as the axial propulsion axis rotation speed increases, the system power consumption significantly increases. The power consumption change trend is consistent with the theory of mixing power and the energy dissipation characteristics of high-viscosity fluids. These results suggest that the CFD model is capable of describing the relative changes in torque distribution and power consumption induced by different differential speed ratios, providing a basis for comparative analysis of flow field characteristics.

It should be noted that direct experimental validation was not performed in the present study due to the difficulty of measuring internal flow characteristics in a large-scale, high-viscosity polyurethane adhesive system. Therefore, the CFD results should be interpreted within the assumptions of the numerical model, including single-phase flow behavior, steady-state approximation, and the selected numerical solution strategy. Further experimental verification, such as torque measurement and scale-down flow visualization, will be considered in future work.

### 2.5. Mathematical Model

The polyurethane adhesive system was simplified as an incompressible single-phase fluid during the mixing process, and its flow behavior was described using a constant-viscosity approximation. Although polyurethane adhesives may exhibit shear-dependent rheological behavior in practical applications, the present study focuses on the influence of differential speed coupling on the overall flow field characteristics and mixing enhancement mechanism. Therefore, an average apparent viscosity was adopted to represent the bulk flow behavior under the investigated operating conditions. The governing equations were based on the conservation of mass and momentum.

The continuity equation is

(1) ∇·u=0

Among them, u represents the velocity vector.

The momentum conservation equation is

(2) $$\rho \left(u\cdot \nabla \right)u=-\nabla p+\mu \nabla^{2}u+F$$

Among them, ρ represents the fluid density, p represents the pressure, μ represents the dynamic viscosity, and F represents the volume force.

To describe the interaction between the multi-axis rotating region and the stationary region, the Multiple Reference Frame (MRF) method is employed to handle the rotating region [21]. The present CFD simulations were performed under a steady-state framework, in which the MRF approach provides an averaged representation of the rotating flow fields and enables comparison of the relative effects of different differential speed ratios with acceptable computational cost. Since the objective of this study is to investigate the influence of differential speed coupling on the overall flow field structure and mixing performance rather than instantaneous blade-passing dynamics, the MRF method was considered appropriate for the present analysis.

In the CFD model, the main dispersing shafts and axial propulsion shafts were defined as rotating regions, while the tank body and other fixed components were treated as stationary regions. The interaction between the rotating and stationary regions was calculated using the MRF approach. The tank wall and shaft surfaces were defined as no-slip boundaries. Since the liquid level was assumed to remain constant during mixing, free-surface deformation was neglected. The residual convergence criterion was set to 10^−5^ for the continuity and momentum equations.

The pressure-velocity coupling is solved using the SIMPLE algorithm. The pressure discretization employs a second-order scheme, while the momentum equations are discretized using a second-order upwind scheme to enhance the numerical calculation accuracy and stability [22].

It should be noted that several assumptions were adopted in the present CFD model. The polyurethane adhesive system was simplified as a single-phase incompressible fluid with constant viscosity, and the shear-dependent rheological behavior, temperature dependence, and complex physicochemical changes during the curing process were not considered. Therefore, the obtained results should be interpreted as a comparative analysis of differential speed effects under the assumed rheological condition, rather than an exact prediction of transient industrial processing behavior. In addition, the Multiple Reference Frame (MRF) method was employed to describe the rotating flow regions, which provides a steady-state approximation of the mixing process. Therefore, the present simulation mainly focuses on the influence of differential speed coupling on the macroscopic flow characteristics and mixing enhancement mechanism. The obtained results are applicable for evaluating the relative performance of different operating conditions, while transient flow fluctuations and dynamic interactions between rotating shafts require further investigation.

### 2.6. Operating Condition Settings

To investigate the influence of differential ratios on the mixing performance of the eccentric four-axis agitation system, the ratio of the axial propulsion shaft speed to the main dispersion shaft speed was used as the control variable. Three typical simulation conditions were established: 50:100, 100:100, and 100:50 rpm, where the first value represents the rotational speed of the axial propulsion shafts and the second value represents the rotational speed of the main dispersing shafts. These conditions correspond to low differential, synchronous rotation, and high differential operating modes, respectively. These three conditions were selected to represent typical differential speed coupling regimes rather than to establish a complete operating optimization map. The objective of this study was to clarify the influence mechanism of differential speed coupling on flow field evolution and mixing characteristics. Therefore, the selected cases were designed to cover representative states from circulation enhancement and synchronous operation to excessive disturbance.

These three conditions were selected to represent three typical differential coupling regimes rather than simply different rotational speeds. The 50:100 condition represents a circulation-dominated mode, where the lower-speed axial propulsion shafts provide continuous bulk circulation while the higher-speed dispersion shafts introduce local shear enhancement. The 100:100 condition represents a synchronous rotation mode with reduced differential interaction between shafts. The 100:50 condition represents a strong disturbance mode caused by a reversed speed relationship, where excessive differential effects may influence flow stability and energy utilization. Therefore, these three cases can provide a comprehensive comparison of the evolution from stable circulation to synchronous operation and excessive flow disturbance.

Among them, the low differential condition was used to analyze the flow field characteristics under a circulation-enhanced coupling mode, where the axial propulsion shaft provided bulk circulation while the main dispersion shaft generated local shear effects. The constant-speed condition was used to study the flow field distribution under the synchronous rotation state of multiple axes; and the high differential condition was used to examine the flow field disturbance and energy transfer characteristics caused by the relatively stronger axial propulsion effect. By comparing the velocity field distribution, streamline structure, power consumption characteristics, and mixing performance differences under different differential ratio conditions, the influence law of differential coupling on the mixing behavior of the high-viscosity polyurethane adhesive system was revealed, and the mixing performance under different differential coupling conditions was comparatively evaluated.

### 2.7. Evaluation Indicators for Hybrid Performance

To comprehensively evaluate the mixing performance of the eccentric four-axis mixing system under different differential ratios, indicators such as unit volume power consumption, velocity variation coefficient (CV), effective mixing area proportion, maximum Lyapunov exponent (LLE), and vortex core coverage rate were selected for comprehensive analysis.

(1) Power consumption per unit volume

The unit volume power consumption is used to characterize the energy consumption level of the system. The smaller the value, the lower the energy required to achieve the mixing of a unit volume of fluid. The formula for calculating the total unit volume power consumption of the system is as follows:

(3) $$P=\sum_{i=1}^{4}P_{i}$$

(4) $$P_{i}=\frac{{2\pi N}_{i}M_{i}}{V}$$

In the formula, P represents the total unit volume power consumption of the system W/m^3^; Pi represents the unit volume power consumption corresponding to the i-th stirring shaft W/m^3^; Ni represents the rotational speed of the shaft in r/s; Mi represents the torque of the shaft in N·m; V represents the effective volume of the stirring vessel in m^3^.

(2) Variance coefficient of speed (CV)

The velocity variation coefficient is used to evaluate the uniformity of the velocity distribution in the flow field. Its calculation formula is

(5) $$CV=\sigma_{v}/\bar{v}$$

In the formula, $\sigma_{v}$ represents the standard deviation of the flow velocity; v is the average velocity of the flow field. The smaller the CV value is, the more uniform the velocity distribution of the flow field is, and the better the mixing effect is.

(3) Proportion of effectively mixed areas

The proportion of the effective mixing area is used to represent the volume fraction of the fluid that participates in the efficient mixing within the stirred vessel. This indicator can reflect the coverage range of the flow field and the overall mixing ability. The larger the value is, the wider the area where the fluid participates in effective mixing is, and the better the overall mixing performance is. In this study, the effective mixing region was defined as the region with sufficient fluid motion, while low-velocity regions with limited material exchange were considered dead zones. The effective mixing region was identified according to the velocity magnitude distribution, and the same criterion was applied for all operating conditions. A velocity magnitude threshold of 0.1 m/s was adopted to distinguish effective mixing regions from weak-flow regions.

(4) Maximum Lyapunov Exponent (LLE)

The maximum Lyapunov exponent is used to quantitatively characterize the chaotic stretching intensity of the flow field [23,24,25]. It describes the exponential separation rate of adjacent fluid trajectories and reflects the stretching capability of chaotic transport processes. A higher LLE value indicates stronger trajectory divergence, fluid stretching, and interface renewal, which are beneficial for material exchange in highly viscous mixing systems. Previous studies have demonstrated that nonlinear dynamic analysis and phase-space reconstruction are effective approaches for identifying flow instability and complex nonlinear evolution in complex fluid systems [26]. These findings provide a theoretical basis for applying nonlinear indicators to characterize complex transport behaviors in mixing processes. Therefore, LLE was introduced in this study to reveal the nonlinear mixing characteristics induced by differential speed coupling. When LLE is greater than zero, adjacent fluid trajectories exhibit exponential divergence, indicating enhanced chaotic stretching characteristics. A larger LLE value represents stronger fluid stretching and folding behavior, which is generally associated with improved material transport and mixing performance [24,27].

The LLE was calculated based on the Lagrangian particle tracking method. A series of virtual particles were uniformly distributed in the mixing domain, and their trajectories were obtained by integrating the local velocity field. The velocity at particle positions was interpolated from the CFD results, and the LLE value was determined from the average divergence behavior of multiple particle pairs. The same particle tracking parameters and calculation procedure were applied to all operating conditions to ensure a consistent comparison.

(5) Vortex core coverage

The coverage of vortex cores is used to describe the spatial distribution range of vortex structures in the flow field. By calculating the proportion of the vortex core area in the entire flow field, it can evaluate the fluid circulation capacity and energy transfer level. A higher coverage of vortex cores usually indicates a stronger coupling effect of the flow field and a more thorough material exchange process. The vortex core coverage was evaluated based on the spatial distribution of vortex structures identified from the flow field, representing the proportion of vortex regions in the whole mixing domain. The effective mixed energy consumption ratio was used to describe the relationship between energy input and the volume fraction of effectively mixed regions.

Based on the above evaluation indicators, a systematic evaluation is conducted on the energy consumption characteristics, flow field uniformity, chaotic mixing ability, and overall mixing performance of the eccentric four-axis stirred system under different differential ratios.

## 3. Results and Discussion

### 3.1. Distribution Characteristics of Velocity Field

The velocity field distribution inside the mixing vessel can directly reflect the fluid circulation ability, the range of shear effect, and the degree of uniform mixing. It is an important basis for evaluating the mixing performance of high-viscosity systems [8,10]. To analyze the influence of the differential ratio on the flow field structure of the eccentric four-axis mixing system, the velocity cloud diagrams of the characteristic sections in XY, YZ, and ZX directions under different working conditions were extracted respectively, and parameters such as the maximum velocity, the inter-axis velocity gradient, the proportion of the effective mixing area, the velocity variation coefficient (CV), and the average velocity near the wall were statistically compared for quantitative analysis.

**Figure 5:**
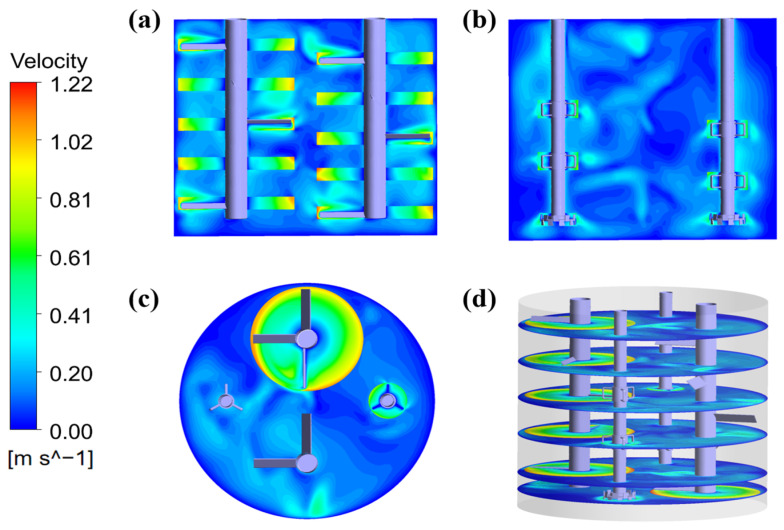
Velocity field distribution of the eccentric four-shaft mixer under the 50:100 differential speed ratio. (**a**) XY section; (**b**) YZ section; (**c**) ZX section; (**d**) comparison of velocity distributions at different sections.

**Figure 6:**
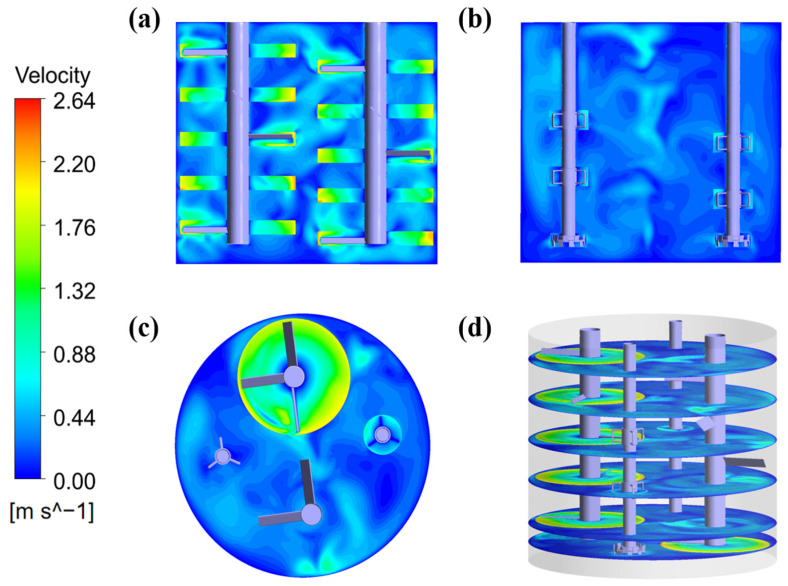
Velocity field distribution of the eccentric four-shaft mixer under the 100:100 synchronous speed condition. (**a**) XY section; (**b**) YZ section; (**c**) ZX section; (**d**) comparison of velocity distributions at different sections.

**Figure 7:**
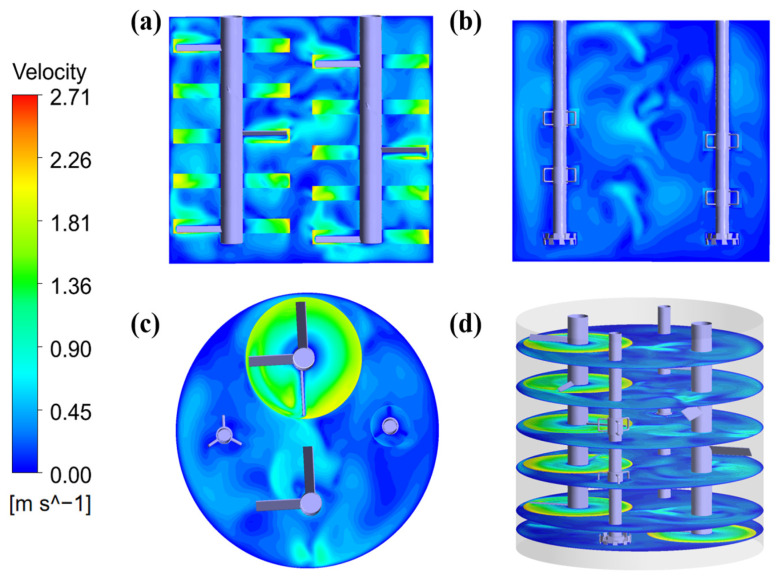
Velocity field distribution of the eccentric four-shaft mixer under the 100:50 high-differential speed condition. (**a**) XY section; (**b**) YZ section; (**c**) ZX section; (**d**) comparison of velocity distributions at different sections.

Figure 5, Figure 6 and Figure 7 and Table 2, it can be seen that the internal flow field structure of the mixing tank varies significantly under different differential ratios. In the low differential condition (50:100), a closed-loop circulation flow field was formed throughout the top, middle, and bottom areas of the tank, allowing the fluid to achieve continuous transportation and exchange throughout the entire container [28,29]. The local high shear zone generated by the main dispersion axis couples with the circulating flow formed by the axial propulsion axis. While ensuring the dispersion of the fillers and the untying of the molecular chains, this effectively enhances the axial transport capacity. As shown in Figure 5 and Table 2, the 50:100 condition forms a relatively uniform closed-loop circulation flow field. The velocity variation coefficient decreases to 0.38, and the effective mixing region reaches 60%, indicating that the stable circulation structure shown in Figure 5 corresponds to improved flow uniformity. This improvement is mainly attributed to the complementary operation of the two types of stirring shafts. Although the rotational speed of the axial propulsion shafts is lower than that of the dispersion shafts under the 50:100 condition, the larger blade-induced pumping effect of the propulsion structure enables effective bulk circulation, while the faster dispersion shafts provide localized shear fields. The differential speed coupling between the two functional units reduces the attenuation of material transport between different regions.

Under the constant-speed condition (100:100), the four stirring shafts operate synchronously, and the flow field presents a relatively obvious symmetrical structure. Due to the mutual impact of the fluids in the inter-axis area, the kinetic energy in some areas is mutually canceled out, and the lateral transport capacity is inhibited. At the same time, the high-viscosity fluid forms a low-speed stagnant layer in the central area, reducing the coverage area of the high-speed flow region. Table 2 shows that the proportion of the effective mixing area in this condition has decreased to 48%, and the speed variation coefficient has increased to 0.55, indicating a significant reduction in the uniformity of the flow field.

Under the high-speed differential working condition (100:50), the propulsion shaft operates at a high speed, generating intense axial circulation. Meanwhile, the rotational speed of the main dispersion shaft is relatively low, making it difficult for the two types of functional flow fields to form effective synergy. The significant speed difference leads to obvious fluid interference in the inter-axial area. The high-speed and low-speed zones alternate, resulting in a decrease in flow field stability. The maximum speed in this condition reaches 2.657 m/s, but the proportion of the effective mixing area is only 35%, and the speed variation coefficient increases to 0.61, which is the highest among the three sets of working conditions. This indicates that simply increasing the local flow velocity cannot improve the overall mixing effect.

From the quantitative indicators, as the differential ratio increases, the maximum flow velocity rises from 1.194 m/s to 2.657 m/s, but the proportion of the effective mixing area decreases from 60% to 35%, and the velocity variation coefficient increases from 0.38 to 0.61. This indicates that a high flow velocity does not necessarily correspond to better mixing performance. For high-viscosity polyurethane adhesive systems, the uniformity of the flow field and the ability to cover the entire area are more important than local high-speed shearing. Moderate differential speed can balance local shearing and overall cyclic effects, allowing the fluid to form a stable and continuous transport network within the reactor, thereby achieving higher mixing efficiency.

**Figure 8:**
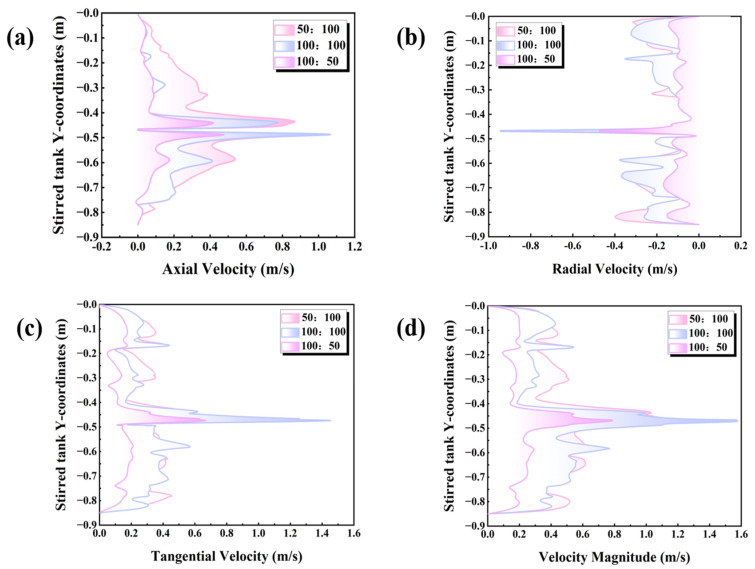
Velocity distribution at different differential ratios. (**a**) axial velocity; (**b**) radial velocity; (**c**) tangential velocity; (**d**) total velocity.

Figure 8 further presents the statistical results of the velocity distribution under different differential ratios. As the differential ratio increases, the proportion of the high-speed area shows an upward trend, but the uniformity of the velocity distribution decreases. The 50:100 condition can achieve a relatively uniform velocity distribution while maintaining a large flow coverage range, which is consistent with the aforementioned effective mixing area and CV analysis results.

Based on the comprehensive velocity cloud diagrams and quantitative evaluation results, the 50:100 condition forms the most uniform and stable global circulation flow field. This result suggests that mixing efficiency in high-viscosity systems is not determined by the maximum local velocity, but by the coordination between flow coverage and energy utilization. Excessive velocity difference may increase local disturbance but simultaneously destroy large-scale circulation structures. It outperforms the other two conditions in terms of the proportion of the effective mixing area and the uniformity of the velocity, and can be regarded as the key research object for subsequent streamline structure and chaotic mixing analysis.

### 3.2. Streamline Structure Analysis

The streamline structure can reflect the transport path and circulation connectivity of the fluid within the stirred vessel, and is an important basis for evaluating the macroscopic mixing performance of high-viscosity systems. To further analyze the influence of the differential ratio on the fluid circulation behavior, the streamline distribution characteristics under different operating conditions were compared and analyzed.

**Figure 9:**
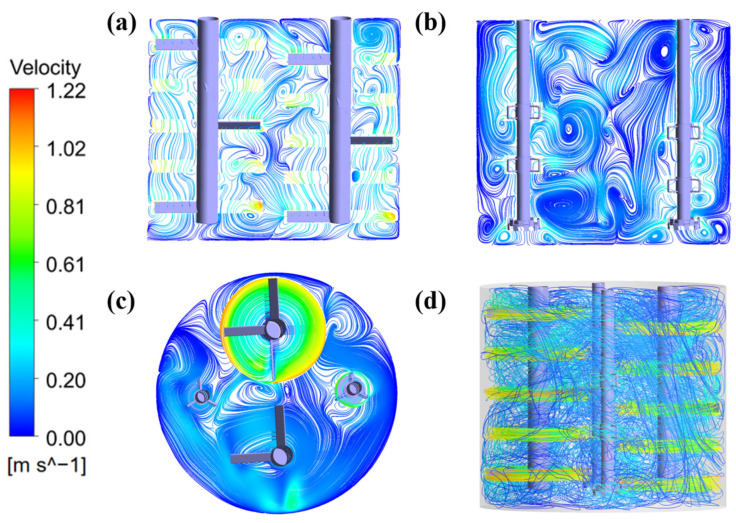
Streamline diagram of 50:100 low-differential speed condition (**a**) XY section; (**b**) YZ section; (**c**) ZX section; (**d**) three-dimensional distribution.

As shown in Table 3 and Figure 9, the 50:100 low differential ratio condition has the best streamline connectivity. Under this condition, the density of the main dispersion axial streamline reaches 15 lines/m^2^, the proportion of the near-wall reflux zone and the proportion of the axial dead zone area are both 0, the density of the bottom streamline in the vessel reaches 8 lines/m^2^, and the proportion of effective mixing energy consumption reaches 72%. This indicates that the fluid in the vessel can form a continuous and stable closed-loop circulation structure. The near-wall area, the axial area, and the bottom area of the vessel can all achieve effective fluid exchange. The local shear flow field generated by the main dispersion axis and the axial circulation formed by the axial propulsion axis are coupled with each other. Under the pressure difference between the axial areas driven by the axial differential ratio, the fluid is promoted to be transported laterally, thereby forming a circulation network covering the entire vessel.

**Figure 10:**
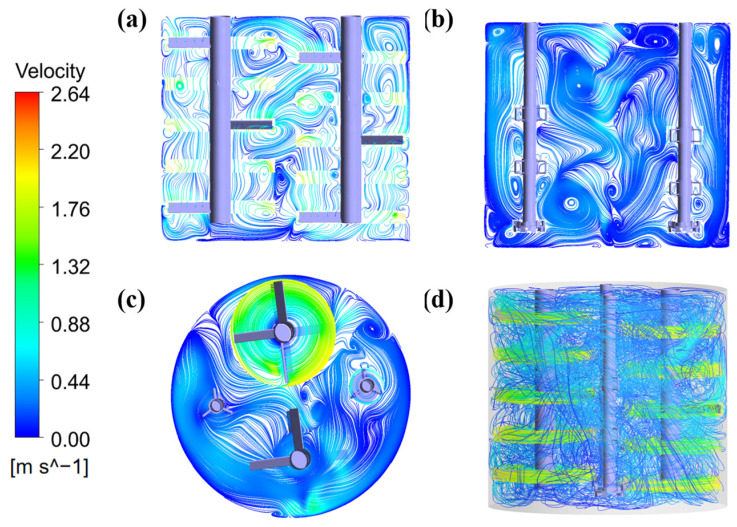
Streamline diagram of 100:100 constant velocity condition. (**a**) XY section; (**b**) YZ section; (**c**) ZX section; (**d**) three-dimensional distribution.

In contrast, as shown in Figure 10, the streamline density under the 100:100 constant-speed condition decreased to 10 lines per square meter, the proportion of the near-wall recirculation zone increased to 5%, the area of the inter-axis dead zone reached 8%, and the proportion of effective mixing energy consumption decreased to 55%. Due to the synchronous operation of each stirring shaft, the flow field exhibited strong symmetry. The symmetric flow pattern limits the exchange between different regions because the fluid mainly follows repetitive circulation trajectories rather than experiencing sufficient stretching and folding. Therefore, although the local velocity remains high, the effective utilization of input energy is reduced. In some areas, fluid counter-flow occurred, which weakened the cross-regional transport capacity and led to flow blockage in local areas.

**Figure 11:**
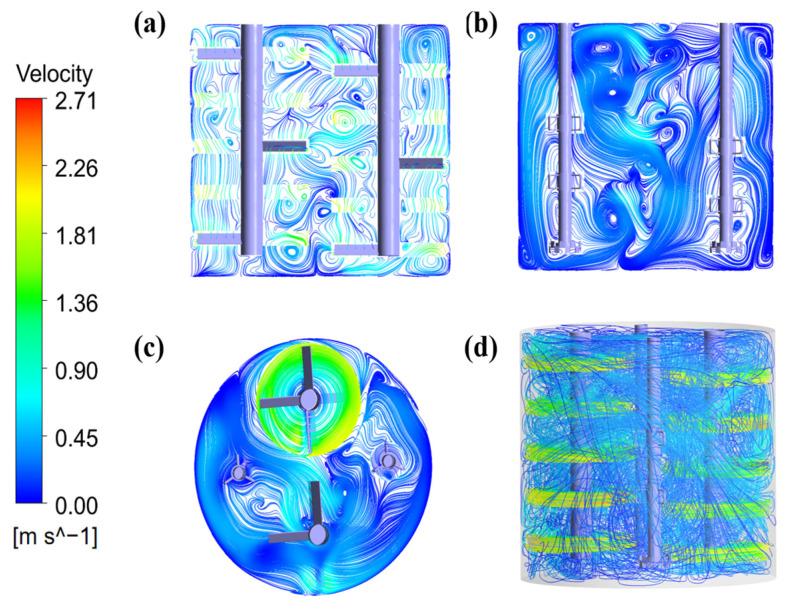
Streamline diagram of 100:50 high-differential speed condition. (**a**) XY section; (**b**) YZ section; (**c**) ZX section; (**d**) three-dimensional distribution.

As shown in Figure 11, under the 100:50 high-speed differential working condition, the streamline structure further deteriorates. The density of the main dispersed axial streamlines is only 5 lines per square meter, the proportion of the near-wall recirculation zone increases to 12%, the proportion of the inter-axis dead zone area reaches 18%, and the density of the bottom streamlines decreases to 3 lines per square meter, with the proportion of effective mixing energy consumption being only 45%. The excessive speed difference leads to enhanced flow field disturbance; some streamlines break and form local recirculation zones, and a large amount of energy is consumed in the ineffective flow process, thereby reducing the overall mixing efficiency.

Overall, the low-differential speed condition can form the most complete fluid circulation network, effectively avoiding near-wall stagnation and inter-stage dead zones, thereby creating favorable conditions for the subsequent formation of vortex structures and chaotic mixing enhancement.

### 3.3. Core Evolution and Chaotic Mixing Mechanism

For high-viscosity polyurethane adhesive systems, due to the low Reynolds number, it is difficult to form a fully developed turbulent structure. The mixing process mainly relies on the suction, stretching, and replacement effects of the vortex cores to achieve material exchange. Therefore, the evolution law of the vortex structure can directly reflect the chaotic mixing ability of the system.

**Figure 12:**
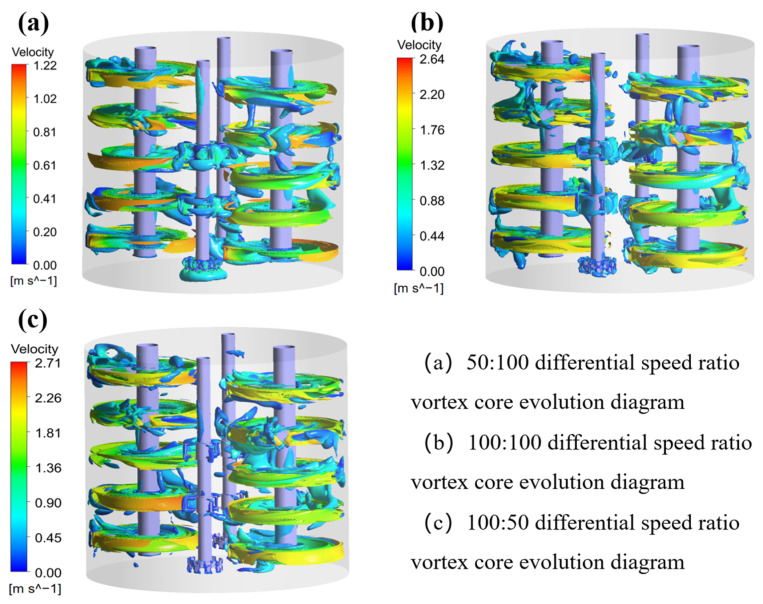
Evolution diagram of vortex cores with different speed ratios.

As shown in Figure 12 and Table 4, the 50:100 low-speed differential condition exhibits the best chaotic mixing characteristics. In this condition, the main vortex core diameter reaches 0.3 m, a coupled large vortex core with a diameter of 0.5 m is formed in the inter-axis area, the vortex strength of the main vortex core reaches 0.8 s^−1^, the coverage range of the vortex core reaches 85%, and the maximum Lyapunov exponent (LLE) reaches 0.018 s^−1^, which is evaluated as strong chaotic mixing.

Moderate differential speed can break the symmetry of the flow field and promote the formation of stable large-scale coupled vortex structures between the axes. The formation of coupled vortices provides a continuous pathway for fluid renewal, which enhances interface deformation and increases the probability of material exchange in high-viscosity systems where molecular diffusion is limited. The coupled large vortex continuously sucks the surrounding fluid during rotation, achieving continuous stretching, folding, and replacement of materials, and simultaneously generating a large number of secondary micro-vortex structures, thereby forming a multi-scale mixing mode of “large vortex strengthening the global circulation, micro-vortex optimizing local dispersion”. This process has typical chaotic convection mixing characteristics [23,24,25,26,27].

Under constant-speed conditions, the chaotic mixing ability is the weakest. Although the diameter of the main vortex core still reaches 0.25 m, no cross-regional coupling large vortex core has been formed. The coverage of the vortex core is only 40%, and the LLE value drops to 0.010 s^−1^. Due to the long-term stable and symmetrical state of the flow field, the fluid mainly undergoes regular revolution motion, lacking the stretching and folding effects required for chaotic mixing; thus, the overall mixing ability is significantly limited [24,27].

Under high-speed differential conditions, the diameter of the main vortex core is less than 0.1 m, the vortex strength is less than 0.2 s^−1^, and no stable coupled large-scale vortex structure is formed. The coverage range of the vortex core is 55%, the LLE value is 0.012 s^−1^, and it shows a medium level of chaotic mixing.

Excessive rotational speed difference leads to the fragmentation of the original large-scale vortex structure, resulting in the formation of a large number of disordered micro-vortices. Although local disturbances are enhanced, the stability of the fragmented vortex structure is poor, making it difficult to achieve long-distance material transportation. Therefore, the overall mixing performance actually decreases.

In conclusion, moderate differential conditions are conducive to the formation of a stable large-scale coupled vortex structure, increasing the chaotic mixing intensity, and thus achieving efficient mixing of high-viscosity systems.

### 3.4. Differential Coupling Enhancement Mechanism

The aforementioned power consumption analysis, velocity field analysis, streamline structure analysis, and vortex core evolution analysis indicate that the differential ratio is the key operating parameter that affects the mixing performance of the eccentric four-axis stirring system. To further reveal the influence laws of differential regulation on the comprehensive performance of the system, a comprehensive evaluation was conducted on the mixing uniformity, energy efficiency characteristics, and chaotic mixing intensity under different differential ratio conditions.

**Figure 13:**
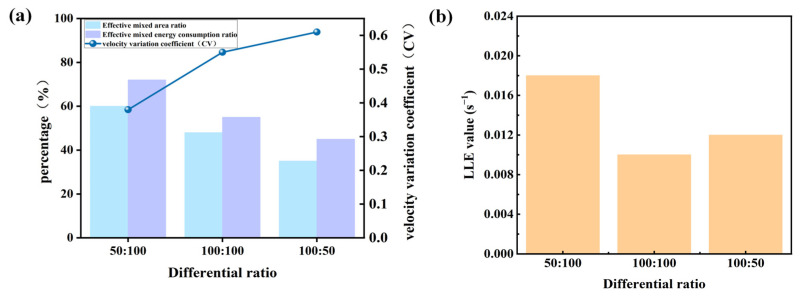
Analysis of gear ratio. (**a**) Effects on the synergistic characteristics of mixing uniformity and energy efficiency; (**b**) quantitative effects on chaotic mixing intensity.

From Figure 13a, it can be seen that the variation in the differential ratio has a significant impact on the homogeneity of the mixture and the energy consumption level. When the low differential ratio condition of 50:100 is adopted, the proportion of the effective mixing area of the system reaches 60%, the speed variation coefficient is only 0.38, and the unit volume power consumption is only 21.2 W/m^3^, demonstrating high mixing homogeneity and a low energy consumption level. As the differential ratio increases to 100:100 and 100:50, although the local flow velocity increases, the flow field uniformity gradually decreases, the inter-axis dead zone and recirculation area increase, and the system power consumption significantly increases, resulting in a decline in the overall energy efficiency level. This indicates that the high-viscosity polyurethane system cannot achieve better mixing effects simply by increasing the speed difference; reasonable differential matching is crucial for maintaining the stability of the flow field and reducing energy consumption.

From Figure 13b, it can be seen that the differential ratio also has a significant regulatory effect on the chaotic mixing intensity. Under the 50:100 condition, a stable large-scale coupled vortex structure is formed, with the vortex core coverage reaching 85% and the maximum Lyapunov exponent (LLE) reaching 0.018 s^−1^, demonstrating the strongest chaotic mixing capability. A moderate differential ratio can break the symmetry of the flow field, promoting the continuous stretching, folding, and rearrangement of fluid micro-tubes during the flow process, thereby improving the material diffusion efficiency. In contrast, the 100:100 condition is difficult to form a continuous and effective chaotic transport process due to the overly stable flow field; the 100:50 condition, due to excessive flow field disturbance, leads to the fragmentation of large-scale vortex structures and a decrease in the chaotic mixing capability.

By combining the results of Figure 13 and the aforementioned analysis, it can be found that the strengthening mechanism of the eccentric four-axis mixing system mainly originates from the differential coupling between the main dispersion axis and the axial propulsion axis [12,13,14]. Among them, the main dispersion axis is responsible for providing a local high shear field, enabling the dispersion and uncoiling of high viscosity systems; the axial propulsion axis is responsible for constructing a global circulation flow field, achieving large-scale material transportation. When the two types of stirring axes operate at a moderate differential speed, a stable large-scale coupled vortex structure can be formed in the inter-axis area, and a circulation transportation network covering the entire reactor can be established, thereby achieving the synergistic enhancement of axial transportation, radial diffusion, and tangential shearing processes.

Therefore, for the 600 L high-viscosity polyurethane adhesive system, the 50:100 differential speed ratio achieves the best balance among mixing uniformity, chaotic mixing intensity, and energy efficiency, and is identified as the optimal operating condition for the investigated eccentric four-shaft stirring system. The results demonstrate that appropriate differential speed regulation can effectively suppress flow field stratification and alleviate mixing deterioration during scale-up of high-viscosity systems, providing theoretical guidance for the structural optimization and operating parameter selection of industrial mixing equipment.

From an engineering application perspective, the proposed eccentric four-shaft configuration provides an effective strategy for large-scale high-viscosity adhesive mixing by integrating high-shear dispersion with efficient bulk circulation. Compared with conventional constant-speed operation, the differential speed coupling strategy offers greater flexibility in controlling energy input and flow field uniformity, thereby reducing the risk of mixing attenuation during scale-up.

### 3.5. Industrial Implications of Differential Speed Regulation

Among the investigated conditions, the 50:100 differential speed condition provides practical guidance for the operation of industrial eccentric four-shaft mixers used for high-viscosity polyurethane adhesives. In this configuration, the main dispersion shafts operating at a higher speed provide sufficient local shear and dispersion capability, while the relatively lower-speed axial propulsion shafts contribute to bulk circulation and material exchange. This coordinated operation improves the balance between local shear enhancement and global circulation, thereby reducing inefficient energy dissipation and minimizing stagnant regions.

From an engineering perspective, the recommended speed ratio can be implemented through independent motor control of the dispersion and propulsion units. The torque and power consumption results obtained in this study can provide preliminary references for motor selection and shaft strength evaluation. For the 600 L mixer investigated, the 50:100 condition exhibited the lowest total power consumption (21.2 W m^−3^) among the studied cases, indicating potential advantages in energy-efficient processing of high-viscosity polyurethane adhesives. Moreover, appropriate speed matching may contribute to better temperature management, reduced air entrainment, and improved product homogeneity during mixing.

However, the direct scale-up of the differential speed strategy should consider additional factors, including material rheology, mixer geometry, filling level, heat transfer characteristics, and production requirements. Therefore, further experimental validation and pilot-scale investigations are required before practical industrial implementation.

### 3.6. Limitations and Future Work

Although the present study provides insights into the differential speed coupling mechanism of the 600 L eccentric four-shaft mixer, several limitations should be acknowledged. First, the polyurethane adhesive system was simplified as a single-phase incompressible fluid, and a constant-viscosity assumption was adopted. In practical applications, polyurethane adhesives may exhibit shear-dependent rheological behavior, and the influence of non-Newtonian characteristics on mixing performance requires further investigation.

Second, the present CFD model was established under a steady-state MRF framework. Although this approach is suitable for comparing relative flow field characteristics under different differential speed conditions, transient effects caused by blade-passing and shaft interactions were not considered. Moreover, chemical reaction, moisture curing, and temperature-dependent viscosity variations during actual adhesive processing were not included.

Third, direct experimental validation of the internal flow field was not performed due to the difficulty of measuring flow behavior in a large-scale high-viscosity adhesive mixer. Therefore, the CFD-based indicators, including effective mixing region, vortex core coverage, and Lyapunov exponent, should be interpreted within the assumptions of the numerical model.

Finally, only three representative differential speed ratios were investigated in this work. The identified favorable condition represents the best performance among the studied cases rather than a universal optimum for all mixer geometries or operating conditions. Future work will focus on experimental verification, non-Newtonian rheological modeling, transient simulations, and broader optimization studies considering operating parameters and mixer configurations.

## 4. Conclusions

This paper focuses on a single-phase system of 600 L high-viscosity polyurethane adhesive. A numerical model of eccentric four-axis stirring was constructed, and the CFD method was employed to study the evolution laws of the flow field and the mixing enhancement mechanism under different differential ratios. Through systematic analysis of power consumption characteristics, velocity field distribution, streamline structure, vortex core evolution, and chaotic mixing characteristics, the following conclusions were obtained.

(1) An eccentric four-axis stirring structure composed of two main dispersion axes and two axial propulsion axes was constructed, achieving the synergy of local high shear dispersion and global circulation transport. The eccentric arrangement can enhance the coupling effect of the flow field between the axes and effectively improve the flow field stratification and local retention phenomena existing in the high-viscosity polyurethane system.

(2) The differential ratio has a significant impact on the flow field structure and mixing performance. Under the 50:100 low differential ratio condition, a closed-loop circulation flow field covering the entire reactor is formed, and the effective mixing area accounts for 60%, with a velocity variation coefficient of only 0.38, demonstrating the best flow field uniformity; the 100:100 constant-speed condition is prone to generate axial dead zones and flow field conflict phenomena; the 100:50 high differential ratio condition leads to an increase in local recirculation zones due to excessive flow field disturbance, resulting in a decline in overall mixing performance.

(3) The analysis of streamline structure indicates that the 50:100 condition has the highest streamline connectivity and energy utilization efficiency. In this condition, the near-wall recirculation zone and axial dead zone are basically eliminated, and the effective mixing energy consumption ratio reaches 72%, enabling the establishment of a stable global circulation transport network and achieving continuous material exchange between different regions.

(4) The analysis of vortex core evolution and chaotic mixing shows that a moderate differential ratio can promote the formation of stable large-scale coupled vortex structures between the axes. Under the 50:100 condition, the vortex core coverage rate reaches 85%, and the maximum Lyapunov exponent (LLE) reaches 0.018 s^−1^, demonstrating the strongest chaotic mixing capability among the investigated cases, which is conducive to the stretching, folding, and interface renewal of fluid micro-tubes, thereby strengthening the material diffusion process of the high-viscosity system.

(5) Based on comprehensive evaluation indicators, including energy consumption, flow field uniformity, circulation transport capacity, and chaotic mixing intensity, the 50:100 speed ratio demonstrated the best overall mixing performance among the investigated differential speed ratios. The results indicate that reasonable differential speed regulation can improve the flow field uniformity and energy utilization efficiency of the investigated 600 L eccentric four-shaft mixer. These findings provide theoretical guidance for further optimization of high-viscosity polyurethane adhesive mixing systems under similar operating conditions.

## Figures and Tables

**Table 1:** Calculation results of torque and power consumption per unit volume for a 600 L single-phase system under different differential speed ratios.

Axial Propulsion Shaft: Main Dispersion Shaft Speed Ratio	Axial Thrust Torque (N · m)	Main Axial Force Distribution (N · m)	Total Unit Volume Power Consumption (W/m^3^)
50:100	0.96	0.15	21.2
100:100	3.70	0.15	68.1
100:50	3.66	0.06	64.2

**Table 2:** Comparison of core quantification parameters of velocity field under working conditions.

Axial Propulsion Shaft: Main Dispersion Shaft Speed Ratio	Maximum Velocity (m/s)	Axial Velocity Gradient (m/s)	Proportion of Effective Mixing Region (%)	Velocity Variation Coefficient (CV)	Near-Wall Average Velocity (m/s)
50:100	1.194	0.25	60	0.38	0.35
100:100	2.590	0	48	0.55	0.45
100:50	2.657	1.86	35	0.61	0.52

**Table 3:** Comparison of core quantitative parameters of streamline under working conditions.

Axial Propulsion Shaft: Main Dispersion Shaft Speed Ratio	Main Dispersed Axial Streamline Density (strip/m^2^)	Percentage of Near-Wall Reflux Zone (%)	Interaxial Dead Zone Area Ratio (%)	Bottom Streamline Density (strip/m^2^)	Effective Mixed Energy Consumption Ratio (%)
50:100 (low differential)	15	0	0	8	72
100:100 (constant speed)	10	5	8	5	55
100:50 (High differential)	5	12	18	3	45

**Table 4:** Comparison of core quantitative parameters of vortex core evolution and chaotic mixing characteristics under three working conditions.

Axial Propulsion Shaft: Main Dispersion Shaft Speed Ratio	Main Vortex Core Diameter (m)	Main Vortex Core Vortex Strength (s^−1^)	Coupled Large Vortex Core Diameter (m)	Vortex Core Coverage Area (%)	LLE Value (s^−1^)	Chaos Mix Level
50:100	0.3	0.8	0.5	85	0.018	Strong Chaos
100:100	0.25	0.65	none	40	0.010	Weak Chaos
100:50	<0.1	<0.2	none	55	0.012	intermediate chaos

## Data Availability

The original contributions presented in this study are included in the article material. Further inquiries can be directed to the corresponding author.
